# The role of integrins in inflammation and angiogenesis

**DOI:** 10.1038/s41390-020-01177-9

**Published:** 2020-10-07

**Authors:** Olachi J. Mezu-Ndubuisi, Akhil Maheshwari

**Affiliations:** 1 Department of Pediatrics, University of Wisconsin School of Medicine and Public Health, Madison, WI USA; 2 Department of Pediatrics, Johns Hopkins University, Baltimore, MD USA

## Abstract

**Abstract:**

Integrins are heterodimeric transmembrane cell adhesion molecules made up of alpha (α) and beta (β) subunits arranged in numerous dimeric pairings. These complexes have varying affinities to extracellular ligands. Integrins regulate cellular growth, proliferation, migration, signaling, and cytokine activation and release and thereby play important roles in cell proliferation and migration, apoptosis, tissue repair, as well as in all processes critical to inflammation, infection, and angiogenesis. This review presents current evidence from human and animal studies on integrin structure and molecular signaling, with particular emphasis on signal transduction in infants. We have included evidence from our own laboratory studies and from an extensive literature search in databases PubMed, EMBASE, Scopus, and the electronic archives of abstracts presented at the annual meetings of the Pediatric Academic Societies. To avoid bias in identification of existing studies, key words were short-listed prior to the actual search both from anecdotal experience and from PubMed’s Medical Subject Heading (MeSH) thesaurus.

**Impact:**

- Integrins are a family of ubiquitous αβ heterodimeric receptors that interact with numerous ligands in physiology and disease. Integrins play a key role in cell proliferation, tissue repair, inflammation, infection, and angiogenesis.
- This review summarizes current evidence from human and animal studies on integrin structure and molecular signaling and promising role in diseases of inflammation, infection, and angiogenesis in infants.
- This review shows that integrin receptors and ligands are novel therapeutic targets of clinical interest and hold promise as novel therapeutic targets in the management of several neonatal diseases.

## Introduction

Integrins are a family of ubiquitous αβ heterodimeric receptors that exist in multiple conformations and interact with a diverse group of ligands. These molecules mediate interactions between cells and of these cells with the extracellular matrix (ECM) and thereby serve a critical role in signaling and homeostasis. By facilitating dynamic linkages between the intracellular actin cytoskeleton and the ECM, integrins also transduce both external and internal mechanochemical cues and bi-directional signaling across the plasma membrane.^1,2^ Integrins are involved in a diverse range of body processes, including cellular survival, inflammation, immunity, infection, thrombosis, angiogenesis, and malignancy. In this review, we highlight the structure and function of integrins; the mechanisms involved in integrin activation and signaling; their role in inflammation, infection, and angiogenesis; and discuss current advances in integrin-targeted therapies. Understanding the factors that regulate integrin structure, function, and signaling would enable us to identify new therapeutic targets.

## Structure of integrins

**Fig. 1:**
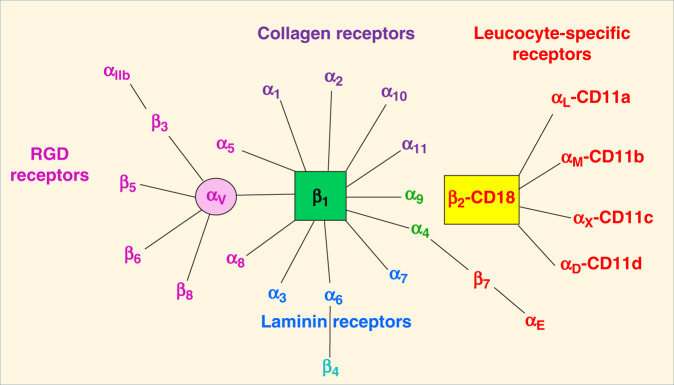
Classification of integrin family. Integrin heterodimers consists of numerous combinations of α and β subunits. With respect to ligand specificity, integrins are generally classified as collagen-binding integrins (α_1_β_1_, α_2_β_1_, α_10_β_1_, and α_11_β_1_), RGD-recognizing integrins (α_5_β_1_, α_V_β_1_, α_V_β_3_, α_V_β_5_, α_V_β_6_, α_V_β_8_, and α_IIb_β_3_), laminin-binding integrins (α_3_β_1_, α_6_β_1_, α_7_β_1_, and α_6_β_4_), and leukocyte integrins (α_L_β_2_, α_M_β_2_, α_X_β_2_, and α_D_β_2_). The β_2_ integrin subunit (CD18) can pair with one of the four α subunits (α_L_-CD11a, α_M_-CD11b, α_X_-CD11c, and α_D_-CD11d), forming leukocyte function-associated antigen-1, Mac1/CR3 (macrophage-1 antigen, complement receptor 3), 150.95/CR4 (complement receptor 4), and CD18/CD11d, respectively. CD11a/CD18 is expressed mainly on all leukocytes, while CD11b/CD18, CD11c/CD18, and CD11d/CD18 are expressed on myeloid cells.^106,107^ The α_M_β_2_ integrin (also known as CR3, CD11b/CD18, or Mac-1) is found on phagocytic cells and implicated in the adhesion of leucocytes to endothelium and opsonization of microbes. Ligands for CR3 include the complement component iC3b, the intercellular adhesion molecule (1CAM-1), and coagulation factors like fibrinogen and factor X.

**Fig. 2:**
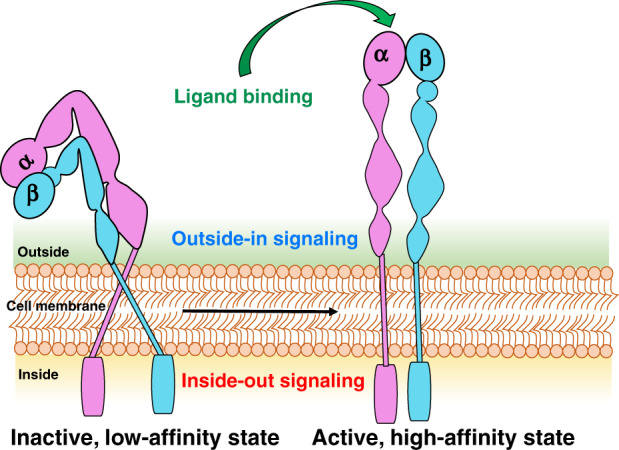
Schematic of integrin structure and activation. Structurally, the αβ integrin subunits are type 1 transmembrane proteins. Each subunit consists of one large multi-domain extracellular segment, one transmembrane helix, and a short cytoplasmic tail. The extracellular region interacts with ECM ligands and is composed of about 1104 (700–1100) residues in the α subunit and 778 residues in the β subunits^32^ and shorter cytoplasmic domains with 30–50 residues.^108^ The short cytoplasmic tails are composed of 20–70 amino acids and mediate interactions with intracellular cytoskeletal and signaling proteins.^1^ In response to intracellular or extracellular stimuli, integrin activation occurs by ligand binding or by the changes on the cytoplasmic domains, resulting in elongation and separation of the legs. Integrins appear in a closed or “bent” conformation on resting cells and display a low binding affinity for ligand rendering them inactive to ligand binding or signal transduction; while once activated, the integrin shape extends to an open conformation leading to a high affinity.^109^ In a closed conformation, integrins show low ligand-binding affinity, partly due to the bend in the center of the α and β subunits, which brings the ligand-binding site within 5 nm of the cell surface.^110^ However, when the conformation is open, the two subunits straighten with increased integrin affinity for the ligand.^111^ The initial binding of extracellular ligand effects separation of the cytoplasmic domains, allowing interaction with signal transduction and cytoskeletal molecules during outside-in signaling, while separation of the cytoplasmic domains by talin and other activators activates the head to enable ligand binding during inside-out signaling.^4^

In mammals, the family of integrins is comprised of 24 αβ pairs of heterodimeric transmembrane adhesion receptors and cell-surface proteins. These pairings are known to involve 18 α and 8 β subunits (Fig. 1),^3^ and their non-covalent associations involve an α and another β subunit (Fig. 2).^4^ The αβ pairings of integrin subunits dictate the specificity of the integrin to a particular ligand, modulate formation of intracellular adhesion complexes, and regulate downstream signaling.^1^ Six α (α_1–6_) and seven β (β_1–7_) subunits are known to form several unique αβ subunit associations (Fig. 1). Interestingly, the earliest discovered integrins, lymphocyte function-associated antigen 1 (integrin α_L_β_2_) and macrophage antigen 1 (integrin α_M_β_2_), derive their specificity from specific α subunits, but these share the same β subunit.^5^

### Integrin α subunit family

The integrin α subunits carry a 200 amino acid “inserted” domain, the I-domain (αI). When present on an integrin, the αI domain is an exclusive ligand-binding site. αI integrins have 13 extracellular domains in 2 subunits, which interact with a variety of ligands. The I-domains are seen in 6 out of the 15 integrin α subunits.

### Integrin β subunit family

In humans, integrin β subunits have a cytoplasmic tail that have <75 amino acids in length, except the β_4_ tail that is about 1000 amino acids long (includes four fibronectin type III repeats).^3^ The integrin β tails have one or two NPxY/F motifs (x refers to any amino acid) that recognize protein modules, phosphotyrosine-binding domains, that are involved in several signaling and cytoskeletal proteins at the cytoplasmic face of the plasma membrane through phosphorylation of the tyrosine (Y) in the NPxY/F motif.^3^ The integrin β subunit family includes β_1–7_, which bind the α subunits in different combinations. The most frequently seen β subunit integrin heterodimers is β_1_. Although β_2_ integrins show functional overlap, the corresponding α subunit defines its individual functional properties.^6^ The β_2_/CD18 chain has also received attention because of its involvement in several inflammatory receptors such as CD11a/CD18, α_L_β_2_, lymphocyte function-associated antigen-1 (LFA-1); CD11b/CD18, α_M_β_2_, Mac-1, complement receptor 3 (CR3); CD11c/CD18 (α_X_β_2_, p150.95, CR4); and CD11d/CD18, α_D_β_2_; Fig. 1). In these β_2_ integrins, the α subunits bind specific ligands such as the intercellular adhesion molecules (ICAMs). The non-I-domain α subunits in other integrins, such as the laminin-binding α_3_, α_6_, and α_7_, and others that recognize the arginine (R), glycine (G), aspartic acid (D) (RGD) motif (α_V_, α_8_, α_5_, and α_IIb_), are also closely related to each other.^7^ The α subunit of each integrin is the primary determinant of its extracellular ligand specificity. The β chain binds acidic residues in ICAMs and in cytoplasmic adapters such as paxillin, talins, and kindlins to facilitate cellular adhesions with the ECM. Integrins interact with the actin cytoskeleton through the talin- and kindlin-binding motifs present in the cytoplasmic domains of their β subunits.^8^

### Characteristics of specific integrin heterodimers

Integrin αβ heterodimers are divided into four classes (leukocyte, collagen-binding, Arg-Gly-Asp (RGD)-binding, and laminin-binding integrins; Fig. 1) based on evolutionary associations, ligand specificity, and restricted expression on white blood cells (β_2_ and β_7_ integrins). *Leucocyte integrins* have a common β_2_ chain that is linked to CD-18 and bind receptors such as ICAM and plasma proteins such as complement components C3b and C4b.^9^ *Collagen-binding integrins* have a common β_1_ chain that binds various α chains in integrins α_1_β_1_, α_2_β_1_, α_10_β_1_, and α_11_β_1_. The α_2_β_1_ integrin binds its primary ligand, collagen,^10^ and chondroadherin, a matrix protein.^11^ The *RGD-binding integrins* have a common α_V_ chain or β_1_ chain. The RGD peptide motif was first discovered in fibronectin^12^ but was later found in several other ECM proteins, such as fibronectin,^9^ osteopontin,^13^ vitronectin,^14^ von Willebrand factor (VWF),^15^ and laminin.^16^ Among the 24 human integrin subtypes known to date, eight integrin dimers recognize the tripeptide RGD motif within ECM proteins, namely: α_V_β_1_, α_V_β_3_, α_V_β_5_, α_V_β_6_, α_V_β_8_, α_5_β_1_, α_8_β_1_, and α_IIb_β_3_. *Laminin-binding integrins* (α_3_β_1_, α_6_β_1_, α_7_β_1_, and α_6_β_4_) mediate the adhesion of cells to basement membranes in various tissues.^9^ The α_4_β_1_, α_9_β_1_, and α_4_β_7_ integrin family binds fibronectin in a RGD-independent manner.^9^

## Integrin–ligand binding and consequent activation

The structure and function of integrins are complex. Integrins bind numerous extracellular ligands, intracellular signaling molecules, and the cytoskeleton in a bivalent-cation-dependent manner with varying specificities. Integrins also have many states with multiple conformations and affinities.

### Mechanism of integrin ligand binding and conformational states

Integrins bind cell-surface ligands to promote cellular interactions with the ECM and with other cells in the transduction of complex signals that modulate many cellular processes, such as adhesion, migration, and differentiation. These soluble, ECM, or cell surface-bound ligands may include growth factors, structural constituents of the ECM, proteases, cytokines, plasma proteins, microbial pathogens, or receptors specific to immune cells. The affinity and avidity of a ligand may change actively by inside-out signaling in specific pathways. Ligand affinity may vary with the strength of interaction and dissociation of a monovalent protein and its ligand, where ligand avidity refers to its ability to form multiple combinations of bonds.^17^

Integrins exist primarily in three conformational states: bent–closed (inactive; the predominant state), extended–closed (active; low affinity or intermediate state), and the extended–open (active; high affinity).^18^ The affinity of integrins to various inhibitory and stimulatory ligands is modulated by bivalent cations, which induce a range of conformational changes in integrins ranging from a folded, inactive, and low-affinity state to a high-affinity conformation (Fig. 2).^19^ These conformational changes in the extracellular domains of integrins modulate both ligand binding and downstream cellular signaling.

### Integrin activation

The activation of integrins increases the affinity of these molecules to extracellular ligands. Integrin tail domains play a critical role in these steps, and any genetic mutations in these parts of integrins can disrupt downstream intracellular signaling.^20^ Integrin-mediated signaling across cell membranes is typically bi-directional and termed “outside-in” and “inside-out” signaling.^20,21^ When integrins interact with ECM ligands, a conformational change allows adherence to downstream adaptor molecules in the cell-membrane plane.^22^ Once clustered, integrins are able to recruit and activate kinases such as Src family kinases, focal adhesion and scaffold molecules such as the adaptor protein p130CRK-associated substrate/breast cancer anti-estrogen resistance 1 (p130CAS/BCAR1).^22^ These integrin-associated complexes include discrete active and inactive integrin organizations, which can activate unique signaling pathways.^23,24^

The extracellular domains of integrins are known to undergo a diverse range of conformational changes that alter the ligand-binding domains. In the cytoplasmic tails of integrins, α-helices are seen as heterodimers,^25^ and the β-strands often bind intracellular proteins, such as talin or filamin.^26,27^ The cytoplasmic tail may undergo several specific conformational changes to bind a range of other signal transducers.^28,29^

### Integrin bi-directional inside-out and outside-in signaling

Mechanical stress^30^ and extracellular chemicals^31^ can induce rapid conformational changes to cause inside-out activation of integrins.^32^ Integrins display bi-directional signaling across the plasma membrane. Ligand binding induces extracellular-to-cytoplasm signal transduction, and inside-out signaling or priming regulates integrin-ligand binding conformations (Fig. 2). During integrin activation and signaling, the cytoplasmic tail acts as both a receptor and transmitter of signals. Specifically, during inside-out signaling, the activating signals make an impression on the cytoplasmic tail to induce large conformational changes to the extracellular domain, thereby transforming the integrin from a resting to an active state.^33^ During outside-in signaling, the binding of a ligand to the extracellular domain of active integrin transmits a conformational change to the cytoplasmic tail, which leads to the activation of kinases and adaptor molecules in the cytosol.^1^ In contrast, talin and kindlin interaction with the β-cytoplasmic tail can trigger inside-out signaling, leading to integrin activation, clustering, and recruitment of intercellular adaptor proteins to strengthen cellular adhesion. Talin is a large dimeric actin-binding protein and a major regulator of integrin activation, and the regulation of talin–integrin interactions is important in the control of integrin activation and signaling pathways.^33^ Direct interactions between the talin head and the short cytoplasmic tails of β integrin subunits disrupt inhibitory interactions between α and β integrin subunits.^33^ This leads to conformational changes in the integrin extracellular domains and consequent increase in their ligand affinity. The role of kindlins are not clearly defined, but they are structurally related to the talin head. The synergistic binding of talin and kindlin to β integrin cytoplasmic tails induces integrin activation by disrupting the α–β interactions at the transmembrane and the cytoplasmic domains.^33,34^

## Integrins in inflammation and infection

In the resting state, β_2_ integrins are expressed specifically on leucocyte receptors. During inflammation, the inflammatory cytokines activate these integrins and promote cellular adherence to the counter-receptors such as ICAMs and promote phagocytosis and cytotoxic killing. Integrin receptors on leukocytes, such as the macrophage-1 antigen (Mac-1, also known as CR3, α_M_β_2_, CD11b/CD18) interact with platelet antigens such as the glycoprotein Ibα (GPIbα) during inflammation. Integrins bind to the pro-domain of transforming growth factor (TGF)-β_1_ to activate it and promote its secretion. The pro-TGF-βs are biosynthesized and stored in tissues in latent forms, and integrins α_V_β_6_ and α_V_β_8_ can uniquely bind and activate pro-TGF-β_1_ and pro-TGF-β_3_. The α_V_β_6_ integrin is known to specifically bind the RGDLXXL/I motif in TGF-β_1_ and TGF-β_3_.^35^

β_2_ integrins promote recruitment of leukocytes to the sites of inflammation by promoting the adhesion of circulating leukocytes to vascular endothelium, transendothelial migration,^36,37^ the formation of immunological synapses in leucocytes,^38^ and inflammatory signaling in involved cells.^39^ Activated β_2_ integrins on dendritic cells (DCs) may act as negative regulators of DC migration in certain conditions and may also regulate T cell activation.^40,41^ β integrins on the leukocyte surface are also involved in the tethering, rolling, and adhesion of leukocytes to activated endothelial cells.^42^ β_2_ integrins can also initiate intracellular signaling pathways in macrophages and neutrophils and stimulate cytokine secretion from these cells either directly or in synergy with Toll-like receptors (TLRs).^43^ Integrins may also integrate the impact of the epidermal growth factor receptor, platelet-derived growth factor receptor, insulin receptor, met receptor superfamily (hepatocyte growth factor receptor), and the vascular endothelial growth factor receptor (VEGFR) in inflammatory cells.^44^

β_2_ integrins are important regulators of adhesion, leukocyte recruitment, and immunological signaling. These integrins mediate adhesive interactions between myeloid cells, endothelial cells, antigen-presenting cells, T cells and the ECM.^45^ L-selectin, the CCR7 chemokine receptor, interacts with specific carbohydrate epitopes on the endothelium and promotes leukocyte rolling and transmigration through the vascular endothelium.^46^ Leukocyte rolling induces a rapid, although transient, increase in the affinity of the β1 and β2 integrins to the endothelial ligands.^47,48^ Conformational changes in the structure of the inserted (I) domain of the α_L_ subunit of LFA-1^49^ enhance firm leukocyte adhesion under shear flow.^31,49^

## Role of integrins in neonatal organs during normal development and inflammation

### Integrins in the lung during normal development and in inflammation

Integrins and receptor tyrosine kinases act with cytokine and growth factors to modulate the extracellular signal-regulated kinase and phosphatidylinositol 3-kinase (PI3K)-AKT signaling pathways during regeneration, inflammation, developmental, and pathological processes in the developing lung.^2,44,50,51^ The ECM in the lung contains collagen, fibronectin, laminin, and entactin,^52^ and alterations in the formation and structure of the ECM during normal development, healing from injury, or in chronic lung disease could lead to profound alterations in the lung structure.^53,54^ For instance, fibronectin in the ECM promotes integrin-mediated cellular migration and differentiation of cells during lung development.^55^ β_1_ integrin activates several signaling pathways, particularly the PI3K/AKT pathway activated during wound healing in the presence of collage VI in the lung ECM.^56^ β_1_ integrins play a critical role in alveolar homeostasis, as seen in chronic lung disease depicted in β_1_ integrin-deficient mice.^57^ In addition, β_1_ integrin-deficient alveolar epithelial cells produce excessive monocyte chemoattractant protein 1 and reactive oxygen species, suggesting that β_1_ integrins may be involved in alveolar homeostasis.^58^ In murine models of bronchopulmonary dysplasia, perinatal exposure to lipopolysaccharide and increased expression of interleukin-33 may activate neutrophils and promote fibronectin degradation in alveolar epithelial cells.^59^ Other studies have noted increased expression of integrin α_2_β_1_ on mast cells and activation/release of inflammatory cytokines.^60,61^ Similar findings have been noted in murine models with *Listeria monocytogenes* infections.^62^ Mice deficient in integrin α_2_^63^ and integrin α_IIb_^64^ show defective platelet interaction with collagen. α_2_β_1_ integrin-null mice have normal angiogenesis but may have altered angiogenic responses during injury repair.^65^ In contrast, integrin β_1_ knockout mice may have altered development and are not viable, indicating an essential role of β_1_ during development. Table 1 outlines murine models of integrins, their target tissues, and signaling.

**Table 1:** Integrin-targeted murine models and the effect of their signal modulation.

Integrin	Tissue target	Effect of signal modulation	Mouse model
α_3_β_1_	Endothelial cells	Inhibition of angiogenesis	Endothelial cells α3−/− knockout mice
α_2_β_1_	Retinal Muller cells	Reduced neovascularization	α_2_β_1_ integrin deficient mice^88^
α_2_β_1_	Mast cells	Cytokine release following Listeria infection	α_2_β_1_ knockout mouse model of Listeria infection^62^
α_V_β_6_	Epithelial cells of the lung	Activates transforming growth factor beta (TGF-β) to regulate pulmonary fibrosis and inflammation	Genetic knockdown^116^
α_V_	Intestinal Th17 cells, colon	Decreased regulatory T (Treg) cells in the colon, leading to severe colitis, autoimmunity, and cancer	α_v_-deficient mice^117^
β_1_	Fibroblasts	Delayed cutaneous wound closure and reduced formation of granulation tissue and reduced ECM production	β_1_-deficient fibroblast-specific knockout mice^118^
β_3_	Fibroblasts, epithelial cells	Accelerated re-epithelialization, enhanced TGF-β signaling, dermal fibroblast infiltration	β_3_-deficient mice (genetic knockdown)^119^

### Integrins in intestinal inflammation and in necrotizing enterocolitis (NEC)

The regulation of intestinal leukocyte responses is vital to maintaining immune homeostasis and prevention of intestinal inflammatory conditions. Integrin α_v_β_5_ is expressed on neonatal intestinal macrophages; the expression is developmentally regulated and is not dependent on microbial colonization. These integrins bind different ECM components, such as laminins, collagens, and fibronectin, and are known to coordinate epithelial cell adhesion and movement.^4,66^ These integrins recognize the RGD tri-peptide sequence present in ECM proteins, such as fibronectin and vitronectin.^67,68^ The integrin α_V_β_5_ can be found in both focal adhesions and in clathrin-coated membrane domains.^69,70^

Integrin α_v_β_8_ plays an important role in epithelial homeostasis and is a major activator of TGF-β expression.^71^ α_3_ and β_1_ integrins, which are known to increase epithelial migration, are upregulated by bacterial products during NEC.^72,73^ TLR4 signaling on enterocytes promotes the efflux of β_1_ integrins from the cytoplasm toward the cell membrane and enhances cell–matrix contacts that limit cellular movement.^74^ In other studies, Besner and colleagues have examined the role of E-cadherin and integrins in NEC and showed that the growth factor, heparin-bound epidermal growth factor, can promote intestinal restitution in NEC through its effects on integrin–ECM interaction and intercellular adhesions.^75^ Intestinal epithelial cells also express α_3_β_1_, another set of integrins of translational importance. In NEC, increased epithelial expression of α_3_β_1_ may impair the migration of epithelial cells needed for mucosal wound healing.^74^ However, the same α_3_ integrins are also required for morphologic differentiation of the intestinal epithelium in the developing intestine.^76^ Despite the physiological needs of the β_1_ integrins, therapeutic targeting of these molecules may still be possible with information on the best timing and the possibility of regionally focused intervention.

### Integrins in the developing eye and in retinopathy of prematurity

**Fig. 3:**
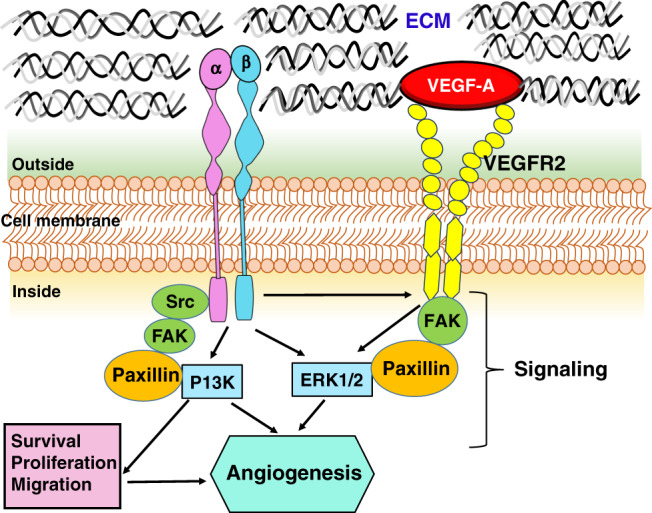
Schematic of integrin regulation of angiogenic signaling. The schematic shows the interaction between the signaling pathways regulated by αβ integrins and the VEGF receptor. VEGF-A promotes angiogenesis through VEGF receptor-2 (VEGFR2), a tyrosine kinase receptor expressed by endothelial cells.^112^ When VEGF-A binds to VEGFR2, numerous intracellular signaling pathways are activated, such as phosphatidylinositol 3-kinase (PI3K), extracellular signal-regulated kinase (Erk), focal adhesion kinase (FAK), c-Src family, and paxillin, a signal transduction adaptor protein associated with focal adhesion.^113,114^ Specifically, FAK phosphorylates its substrate, paxillin, which activates ERK signaling.^114^ When integrins activate the tyrosine phosphorylation of FAK, it binds to signaling structural proteins, PI3K, and paxillin.^115^ Src family kinases (SFKs) play a critical role in cell adhesion, survival, and angiogenesis, interact with VEGF receptor, regulate gene expression of angiogenic growth factors, modulate cell proliferation via the mitogen-activated protein kinases (MAPK)-ERK pathway, and interact with integrins to regulate cell adhesion and migration. ECM extracellular matrix, VEGF vascular endothelial growth factor.

In the developing eye, disruption of the oxygen supply to the retina can disrupt neuronal dysfunction needed for transduction and transmission of photosensitive visual signals to the occipital lobe and other cognitive centers. Integrin α_2_β_1_ and VEGF interact closely in several intracellular angiogenic signaling (Fig. 3).^77,78^ Cyclic peptides selectively inhibit α_V_β_3_ and α_V_β_5_, and are potent inhibitors of endothelial cell invasion and differentiation induced by VEGF-A or fibroblast growth factor-2.^78^ Integrin α_V_β_3_ works synergistically with VEGF to activate angiogenesis in endothelial cells via VEGFR‐2 phosphorylation.^79^ Endothelial cells are the primary cells expressing both VEGFR-2 and α_2_β_1_ integrin.^80^ Proteoglycans such as decorin and perlecan in the ECM of the eye can modulate α_2_β_1_ and play a vital role in angiogenesis.^80^ The C-terminal fragment of perlecan, known as endorepellin, has an opposite effect and blocks angiogenesis through antagonism of VEGFR-2 and α_2_β_1_ integrin on endothelial cells.^80^ Retinal pigment epithelial cells express beta‐8 integrin at the surface, and the knockdown of beta-8 integrin significantly decreased retinal pigment epithelial cell migration in wound-healing assays.^81^

The retinal tissue has one of the body’s highest metabolic demands, placing it at risk of injury from oxidative stress, metabolic derangements, and consequent pathologic neovascularization seen in retinopathy of prematurity (ROP) and other proliferative retinal vitreoretinopathies. ROP is a bi-phasic disease of retinal vascular development due to dysregulation of VEGF.^82,83^ In phase 1, VEGF is downregulated during exposure to hyperoxia, while in phase 2, VEGF is upregulated in relative/true hypoxia. VEGF is known to have several isoforms; VEGFA165 is the predominant isoform in the eye with multiple pro- and anti-angiogenic splice variants.^84^ In a newborn mouse model of oxygen-induced retinopathy (OIR), oxidative stress from fluctuating hyperoxia and hypoxia leads to altered vascular development with tortuous arteries, dilated veins, and capillary attrition, akin to human ROP.^82,85^ These changes persist in adult mice with long-term abnormalities in vascularization, structure, and function both in vivo and histologically.^86,87^

Integrin-targeted therapy holds promise in ROP. Targeting α_2_β_1_ integrin expression on endothelial cells mitigates OIR,^88^ and the administration of 3-[3-(6-guanidino-1-oxoisoindolin-2-yl) propanamido]-3-(pyridin-3-yl) propanoic acid dihydrochloride, a novel non-peptide αvβ3 antagonist, can inhibit retinal neovascularization.^89^ There are exciting possibilities that endothelial α_2_β_1_ may be therapeutic target in pathological angiogenesis.

## Integrins in thrombosis and fibrosis

Platelet adhesion and signaling play key roles in hemostasis and thrombosis. Two platelet receptors, integrin α_IIb_β_3_ and GPIbα, mediate the early and mid-stages of platelet adhesion in the vascular environment.^90^ GPIbα is a key part of the receptor for VWF, and its binding to VWF enables platelet rolling during the formation of thrombotic plugs at the sites of vascular injury.^91–93^ αIIbβ3 is expressed on both platelets and the endothelium, and upon activation, it promotes platelet adhesion and aggregation by cross-linking with soluble fibrinogen, fibronectin, and VWF.

In alloimmune thrombocytopenia, autoantibodies are frequently seen against integrin β_3_ and GPIbα.^94,95^ Intracranial hemorrhages may be seen more frequently in infants with anti-β_3_ integrin antibodies than in those with antibodies against GPIbα.^96^ Existing in vitro and in vivo data suggest that the β_3_ integrin may bind a wider range of ligands, including fibrinogen and VWF, and autoantibodies that block its function may induce a deeper functional deficit than the anti-GPIbα antibodies.^97^

## Integrin-targeted therapies

Integrin dysregulation is implicated in the pathogenesis of numerous diseases with altered angiogenesis, inflammation, or in infectious diseases. In these conditions, therapeutic strategies may either directly target integrins or their ligands. Out of the 24 known human integrins, many have already been identified as therapeutic targets for monoclonal antibodies, peptides, and/or small molecules. In adult subjects, efforts are ongoing to target platelet integrin α_IIb_β_3_ to prevent thrombotic complications after percutaneous vascular interventions, lymphocyte α_4_β_1_ and α_4_β_7_ integrins in the treatment of multiple sclerosis, and β_7_ integrins (α_4_β_7_ and α_E_β_7_ integrins) in inflammatory bowel disease.^98^ Specifically, a humanized anti-α4 antibody (Natalizumab) works in reduction of inflammation in multiple sclerosis by blocking the α_4_β_1_–vascular cell adhesion molecule interaction or the α_4_β_7_–mucosal addressin cell adhesion molecule interaction on mucosal endothelium and blocking leukocyte trafficking across the blood–brain barrier.^99^ In another study, a micellar delivery vehicle decorated with an anti-angiogenic peptide has been shown to inhibit α_V_β_3_-mediated neovascularization in endothelial cells.^100^ Several anticancer drugs have also been developed against integrin ligands or by using integrin-targeted encapsulated nanoparticles as vehicles to unload drugs into the vasculature of several tumors.^101^ In a mouse model of hepatic fibrosis, cyclic peptide-guided liposomes preferentially targeted the activated hepatic stellate cells (not quiescent ones) to treat the fibrotic phenotype.^102^ α_V_β_3_ antagonists are being tried for the inhibition of retinal neovascularization and may have therapeutic value in ROP.^89^ In a mouse model of laser-induced choroidal neovascularization, intravenous injection of irradiated nanoparticles loaded with doxorubicin allowed nanoparticle accumulation in the neovascular lesions and reduced the size of neovascular lesions.^103^ Integrin antagonists may also be used in fibrotic diseases; IDL-2965 is being studied as a selective, highly potent, anti-fibrotic integrin antagonist in idiopathic pulmonary fibrosis.^104^ Small molecule pure antagonists, TDI-4161 and TDI-3761, have been designed to inhibit α_V_β_3_-mediated cell adhesion to α_V_β_3_ ligands.^105^ Further studies are needed to improve the specificity of anti-integrin drugs to improve both the safety profile and therapeutic success of these agents.

## Conclusions

Enhanced understanding of integrin ligand interactions will enable development of therapies targeting specific receptors in order to modulate angiogenic, thrombotic, infections, and inflammatory disorders. Although numerous animal studies have shown promise in the clinical use of integrins as therapeutic targets, there is a need for clinical studies to confirm efficacy and safety in neonates and young infants. In this review, we have summarized and outlined the roles of integrins in inflammation, angiogenesis, and infectious conditions. Therapies could be targeted specifically to alpha subunits, but their overlapping roles are a critical factor to be considered. Further studies are needed both on molecular signaling and regulatory mechanisms of integrin function and the safety and efficacy in clinical settings.
